# From prescription to resistance: integrating one health perspectives into dental antibiotic stewardship in Saudi Arabia

**DOI:** 10.3389/fcimb.2026.1781988

**Published:** 2026-03-25

**Authors:** Anas B. Alsalhani, Bassel Tarakji, Faisal Mehsen Alali, Azza Sioufi, Nasser Raqe Alqhtani, Abdullah Saad Alqahtani, Abdullah Bin Nabhan, Faisal S. Alhedyan, Yousef Alkhaibari, Abdullah Almansour, Hanadi Ghurmallah Alzahrani, Fahad Mubarak Alqahtani, Rayan Awadh Alaonzy, Mohammad Zakaria Nassani

**Affiliations:** 1 Department of Dentistry, Vision Colleges, Riyadh, Saudi Arabia; 2 Department of Histology and Pathology, Faculty of Dentistry, University of Hama, Hama, Syria; 3 Department of Oral Maxillofacial Surgery and Diagnostic Sciences, College of Dentistry, Prince Sattam Bin Abdulaziz University, Al Kharj, Saudi Arabia; 4 Department of Obstetrics and Gynecology, Aljazeera Hospital, Riyadh, Saudi Arabia; 5 Department of Preventive Dental Sciences, College of Dentistry Prince Sattam Bin Abdulaziz University Al Kharj, Al Kharj, Saudi Arabia; 6 Prince Sattam Bin Abdulaziz University, College of Dentistry, Al Kharj, Saudi Arabia; 7 Department of Restorative and Prosthetic Dental Sciences, College of Dentistry, Dar Al Uloom University, Riyadh, Saudi Arabia

**Keywords:** antibiotic stewardship, antimicrobial resistance (AMR), dental antibiotic prescribing, dentists, one health factors, Saudi Arabia

## Abstract

**Background:**

Antimicrobial resistance (AMR) is an escalating global threat, and dentists significantly contribute to antibiotic misuse. In Saudi Arabia, limited national data exist on the appropriateness of dental antibiotic prescribing and the impact of behavioral and One Health factors from prescribing practices.

**Objectives:**

This study aimed to assess the prescribing practices of dentists across Saudi Arabia, identify predictors of adherence to antibiotic prescribing guidelines, and explore awareness of emerging AMR drivers such as climate and diet.

**Methods:**

A national cross-sectional survey was conducted among 905 practicing dentists. The structured questionnaire assessed knowledge, prescribing behaviors, and awareness of antimicrobial resistance, prophylactic use, and One Health factors (climate and diet). Good prescribing practice was defined as correctly answering ≥60% of clinical scenarios. Prescribing appropriateness was evaluated according to established international evidence-based clinical practice guidelines, including recommendations from the American Dental Association (ADA), the American Heart Association (AHA), and the European Federation of Periodontology (EFP), as cited in the manuscript. Descriptive statistics and logistic regression analyses were used to identify predictors of appropriate prescribing.

**Results:**

The results show that only 39.6% adhered to guideline-supported antibiotic prescribing. Adherence was highest in restorative scenarios but notably poor in surgical and prophylaxis conditions. Only 5.5% assessed plaque before prescribing, and less than 10% correctly applied prophylaxis for infective endocarditis. Younger age and female gender were positively associated with appropriate prescribing. Awareness of climate and dietary influences on AMR was low (4.5% and 5.9%, respectively).

**Conclusion:**

Despite high awareness of antimicrobial resistance (AMR), inappropriate antibiotic prescriptions remain prevalent among dentists in Saudi Arabia. This study highlights the need for continuous review and enforcement of dental antibiotic guidelines. By integrating One Health perspectives, it underscores that AMR is influenced not only by clinical practice but also by environmental and behavioral factors such as climate and diet. These findings emphasize the importance of interdisciplinary stewardship interventions and targeted educational initiatives to promote rational antibiotic use within dental care.

## 1 Introduction

The global health crisis of antimicrobial resistance (AMR) continues to escalate, driven in part by the inappropriate use of antibiotics across various healthcare settings, including dentistry (Prestinaci et al., 2015; Farzinnia et al., 2025). Dentistry accounts for a significant proportion of unnecessary antibiotic use globally, and Saudi Arabia is no exception (Alzahrani et al., 2020). Emerging evidence has implicated inappropriate dental antibiotic prescriptions as a contributing factor to the Kingdom’s growing AMR burden (Iyer et al., 2025). While antibiotics remain essential for managing odontogenic infections, they are frequently prescribed for non-bacterial or self-limiting conditions, a trend observed across multiple regions of the country. For instance, a Riyadh-based investigation reported the prescription of antibiotics for clinically unjustified indications such as irreversible pulpitis and routine tooth extractions (Baskaradoss et al., 2018). Similar findings from Jeddah confirm widespread deviations from evidence-based prescribing guidelines (Al-Johani et al., 2017). Compounding the issue, data from the Albaha region revealed a direct association between inappropriate dental prescribing and localized patterns of AMR (Alzahrani et al., 2020).

A core challenge lies in the gap between knowledge and practice. While dentists may be aware of antimicrobial stewardship (AMS) principles, translating this knowledge into clinical behavior remains inconsistent. A nationwide survey revealed that although most Saudi dentists were familiar with the tenets of antibiotic stewardship, their application varied widely, influenced by factors such as professional experience, work environment, and access to training (Alamri et al., 2025a). Alarmingly, even those with good knowledge of prescribing guidelines exhibited suboptimal adherence in practice particularly among those working in the private sector. Furthermore, inappropriate antibiotic use is often exacerbated by patient-related behaviors, such as early discontinuation of treatment, which further undermines therapeutic outcomes and increases resistance risk (Alamri et al., 2025b).

Additional studies across major cities in Saudi Arabia have highlighted systemic educational gaps, exposing deficiencies in dentists’ understanding of AMR and contributing to regional variations in prescribing patterns (Osailan et al., 2021). Earlier investigations echo these concerns and advocate for targeted stewardship interventions (Al-Harthi et al., 2013). Even within specialized fields such as implantology, antibiotic prescribing for infection prophylaxis is often inconsistent and lacks alignment with international recommendations (El-Kholey et al., 2018).

Despite national progress in developing clinical guidelines, significant barriers to implementation persist. Evidence from the Eastern Province demonstrates that dentists’ self-reported awareness does not reliably correlate with their actual prescribing behavior (Al-Johani et al., 2017). Moreover, findings from a national survey underscore a pronounced sectoral divide: while public-sector practitioners tend to align more closely with established guidelines, private-sector dentists show markedly lower adherence (Iyer et al., 2025). This points to institutional culture as a key determinant of prescribing decisions and highlights the need for policy interventions tailored to specific healthcare settings.

The persistence of regional and temporal inconsistencies in dental antibiotic prescribing underscores the urgency of sustained educational reform, curriculum enhancement, and the national implementation of antimicrobial stewardship programs. Within the strategic framework of Saudi Arabia’s Vision 2030, which prioritizes healthcare quality and innovation, there exists a timely opportunity to institutionalize AMS training in undergraduate and continuing dental education. Equally important is the need to raise patient awareness about the responsible use of antibiotics to mitigate the escalating threat of AMR.

This study seeks to support national antimicrobial stewardship (AMS) efforts by offering a comprehensive evaluation of antibiotic prescribing practices among dentists in Saudi Arabia. Utilizing a structured, multi-domain questionnaire, the research assesses practitioners’ knowledge and adherence to evidence-based guidelines across a range of common clinical scenarios including prophylactic, periodontal, surgical, endodontic, and restorative contexts. The study further examines the influence of key contextual factors, such as workplace sector (public versus private) on prescribing behaviors. In addition, it explores dentists’ awareness of antimicrobial resistance (AMR) and their perceptions regarding the need for standardized national prescribing protocols. By identifying critical gaps between knowledge and clinical practice, this research aims to generate evidence that informs AMS policy development and promotes the rational use of antibiotics within dental care settings across Saudi Arabia.

## 2 Materials and methods

### 2.1 Study design and ethical considerations

This study utilized a descriptive, cross-sectional survey design to evaluate antibiotic prescribing patterns and adherence to clinical guidelines among dentists practicing in the Kingdom of Saudi Arabia. The study specifically aimed to explore dentists’ prescribing behaviors across diverse clinical scenarios, including prophylactic, periodontal, surgical, endodontic, and restorative cases, as outlined in the structured questionnaire. Data were collected between April 2024 and February 2025 using a self-administered online questionnaire created via Google Forms.

The survey link was distributed to dental practitioners across multiple social media platforms including WhatsApp, X (formerly Twitter)- to promote broad participation from dentists across various regions of Saudi Arabia using a self-administered online questionnaire designed on Google Forms. The target population consisted of current practitioners in both public and private healthcare sectors within the Kingdom. All participants were informed of the study’s objectives, and electronic informed consent was obtained before participation. Inclusion criteria required participants to be actively engaged in clinical practice in Saudi Arabia during the study period. Responses that failed to meet the inclusion criteria were excluded to maintain data quality and reliability.

The study adhered to internationally recognized methodological and reporting frameworks to ensure rigor, transparency, and reproducibility. Specifically, it was conducted and reported in accordance with the Strengthening the Reporting of Observational Studies in Epidemiology (STROBE) statement, which provides comprehensive guidance for the design, implementation, and reporting of observational research, including elements such as study setting, participant selection, data sources, variables, bias management, and analytical methods (von Elm et al., 2007). Furthermore, the study’s online survey component was designed following the Checklist for Reporting Results of Internet E-Surveys (CHERRIES), which ensures methodological robustness and transparency in web-based data collection, covering critical aspects such as participant recruitment, informed consent, and data protection procedures (Eysenbach, 2004). Together, adherence to these frameworks enhanced the methodological soundness and reliability of findings derived from this national dental survey.

Ethical approval for this study was granted by the Research Ethics Committee of Prince Sattam bin Abdulaziz University, Al Kharj, Saudi Arabia (Reference No. SCBR-436/2025). The research adhered to the Declaration of Helsinki and national ethical standards. Participation was voluntary, and anonymity was guaranteed, with no personal identifiers collected or stored.

### 2.2 Setting and sample

The study involved dental practitioners engaged in clinical practice, including both general dentists and specialists. However, the survey did not classify participants based on their specific areas of specialization. The research was conducted solely in Saudi Arabia and focused on dental practitioners employed in both public and private healthcare sectors. The survey aimed to capture a representative sample reflecting variations in gender, age, and professional sector across various regions in Saudi Arabia, including Riyadh, Jeddah, Dammam, Albaha, and other provinces. The One Health concept was assessed through targeted survey items evaluating participants’ awareness of (1) the relationship between climate change and antimicrobial resistance, (2) the potential influence of dietary patterns on antimicrobial resistance, and (3) behavioral determinants such as routine plaque assessment prior to antibiotic prescription.

According to the latest data from the Ministry of Health (MOH) Statistical Yearbook, in 2023 (Official government website of the Government of the Kingdom of Saudi Araba, 2023), there were 25,970 dentists in Saudi Arabia across both public and private sectors. This number includes 10,800 female dentists and 15,170 male dentists, spread across 17 major regions of the country. Using OpenEpi (Version 3.01), with a 99% confidence level, a 5% margin of error, and a 50% response distribution, the minimum sample size required for this study to ensure it is representative is 664 participants.

To enhance statistical power and generalizability, data collection continued until at least 900 valid responses were obtained.

To ensure data quality and validity, only fully completed and verified responses were included in the final analysis.

### 2.3 Questionnaire development

The survey instrument was rigorously developed to assess the clinical prescribing behaviors of dentists in Saudi Arabia. It consisted of 23 clinical scenarios organized into five antibiotic prescribing domains: prophylactic, periodontal, surgical, endodontic, and restorative, in addition to a demographic and professional information section. These domains were designed to reflect the multifaceted dimensions of antibiotic use in dental practice and were adapted from validated national and international instruments (AboAlSamh et al., 2018; Baskaradoss et al., 2018; Abraham S et al., 2020; Alzahrani et al., 2020; Abdellatif et al., 2025). Prescribing appropriateness for each clinical scenario was evaluated according to established international evidence-based clinical practice guidelines, including recommendations from the American Dental Association (ADA) and associated clinical guidance (Lockhart et al., 2019; Tampi et al., 2019), the American Heart Association (AHA) recommendations for infective endocarditis prophylaxis (Thornhill et al., 2022), and the European Federation of Periodontology (EFP) S3-level clinical practice guidelines (Sanz et al., 2020). These guidelines served as the reference standard for determining whether antibiotic use was indicated or non-indicated in each scenario. The scoring approach used to evaluate prescribing quality was also clearly defined. Good antibiotic prescribing practice was determined based on Bloom’s modified cut-off, with participants classified as demonstrating good practice if they correctly answered ≥60% of the prescribing scenarios (≥14 of 23 items). The first section, General and Professional Information, captured demographic and contextual data, including age, gender, and work sector (public or private). It also assessed preferred antibiotic classes (such as penicillins, macrolides, cephalosporins, and fluoroquinolones). The participants in this section also asked about their perceived need for dentistry-specific antibiotic guidelines, awareness of antibiotic resistance, the impact of climate change on antibiotic overuse or resistance, and the influence of diet on antibiotic resistance.

The second section addressed prophylactic antibiotic prescribing behaviors across a range of procedures, including endocarditis-risk scenarios, third molar extractions (non-impacted and semi-impacted), dental implant placement, hard tissue augmentation (guided bone regeneration), and mucogingival surgeries.

The third section focused on periodontal conditions, asking about antibiotic prescribing for cases such as necrotizing periodontal disease, chronic periodontal disease, necrotizing ulcerative stomatitis, gingivitis, periodontal abscesses, and peri-implantitis. It also evaluated whether dentists performed plaque assessments before initiating antibiotic therapy, an important step in evidence-based periodontal management. The fourth section explored prescribing practices for common surgical conditions, namely pericoronitis and dry socket (alveolar osteitis).

The fifth section investigated the antibiotic prescriptions in endodontic cases, including acute and chronic apical abscesses, symptomatic irreversible pulpitis, reversible pulpitis, and asymptomatic periapical periodontitis. Evidence-based practice recommends antibiotic use only for acute abscesses with systemic involvement; the survey captured adherence or deviation from this guidance. The sixth section addressed restorative dentistry, assessing whether respondents routinely prescribed antibiotics prophylactically or post-invasive operative procedures, and whether they use it as a preventive measure before restorative treatment. Current guidelines discourage routine antibiotic use in restorative procedures unless specific risk factors are present.

To ensure clarity, cultural appropriateness, and clinical relevance, the questionnaire was reviewed by two senior consultants specializing in Oral and Maxillofacial Surgery and Preventive Dental Sciences. Their evaluation focused on content relevance, alignment with current antibiotic prescribing guidelines, and suitability for the Saudi dental practice context. In addition to expert revision, a pilot study was conducted with 10 dentists to evaluate the instrument’s face validity, logical flow, internal coherence, and ease of use in an online format. Minor adjustments were made based on consultants’ recommendations and pilot study feedback to improve question clarity, refine terminology, and standardize response options. The finalized questionnaire was validated and implemented for data collection. Internal consistency was evaluated separately for each prescribing domain within the Saudi sample. The results demonstrated good reliability for the surgical (α = 0.734) and restorative (α = 0.777) domains, reflecting satisfactory internal consistency in these sections. In contrast, the periodontal and endodontic domains showed lower internal consistency, which may be attributed to the broader clinical scope and variability of the prescribing scenarios encompassed within these categories.

### 2.4 Data collection procedure

The data collection period extended from April 2024 to February 2025, encompassing both public- and private-sector dentists.

Participation was voluntary and anonymous, and all respondents were required to provide electronic informed consent before accessing the questionnaire. To promote response completeness, all items were set as mandatory fields. The online survey process adhered to the Checklist for Reporting Results of Internet E-Surveys (CHERRIES) to ensure methodological rigor and data integrity.

Following data submission, responses were screened for accuracy, completeness, and duplication. Entries that were incomplete, inconsistent, or submitted multiple times were automatically excluded based on timestamp and IP verification. The final dataset comprised only validated, fully completed responses, ensuring high-quality and reliable data for analysis in accordance with STROBE recommendations for observational studies.

### 2.5 Data analysis

Data were analyzed using IBM SPSS Statistics for Windows, Version 25.0 (IBM Corp., Armonk, NY, USA). Before analysis, responses were screened for completeness, and invalid entries were removed to ensure data quality. The final analytic sample consisted of 905 valid responses from dentists practicing across the Kingdom of Saudi Arabia.

Descriptive statistics including frequencies and percentages were used to summarize participants’ demographic characteristics, professional profiles, antibiotic prescribing patterns, and awareness of antimicrobial stewardship principles. These analyses provided an overview of the distribution of responses across the major prescription domains (prophylaxis, periodontal conditions, surgical conditions, endodontic cases, and restorative care).

Good antibiotic prescribing practice was defined as correctly answering ≥60% (≥14 of 23 items) in the antibiotic prescription domain. This threshold was based on Bloom’s modified cut-off criteria, which have been widely adopted in recent studies evaluating knowledge and practice related to antibiotic use and antimicrobial resistance (Najjar and Hassan, 2021; Alzahrani et al., 2025), and is consistent with widely accepted standards for determining performance levels in educational and behavioral assessments (Sullivan, 2011).

Associations between dentists’ characteristics and the quality of antibiotic prescribing behavior were examined using Chi-square (χ²) tests. Independent variables included age group, gender, work sector (public vs. private), and perceived need for dentistry-specific antibiotic guidelines.

A multivariate binary logistic regression model was created to identify independent predictors of good antibiotic prescribing practice. Predictor variables in the model included age, gender, work sector, and perceived need for dentistry-specific antibiotic guidelines. Regression results were reported as odds ratios (ORs) with corresponding 95% confidence intervals (CIs). Statistical significance was set at p < 0.05, and all tests were two-tailed.

**Figure 1:**
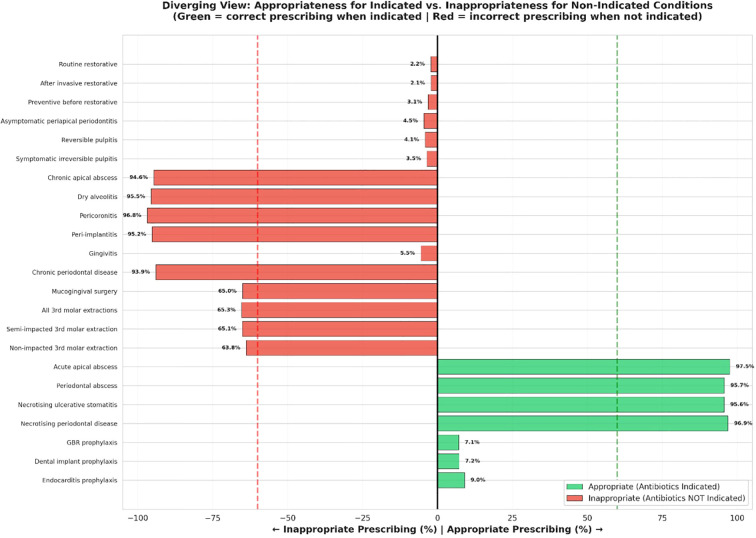
Diverging view of appropriateness for indicated versus inappropriateness for non-indicated conditions. The balance of correct and incorrect antibiotic prescriptions across various dental scenarios, illustrating the appropriateness for indicated conditions and inappropriateness for non-indicated conditions.

**Figure 2:**
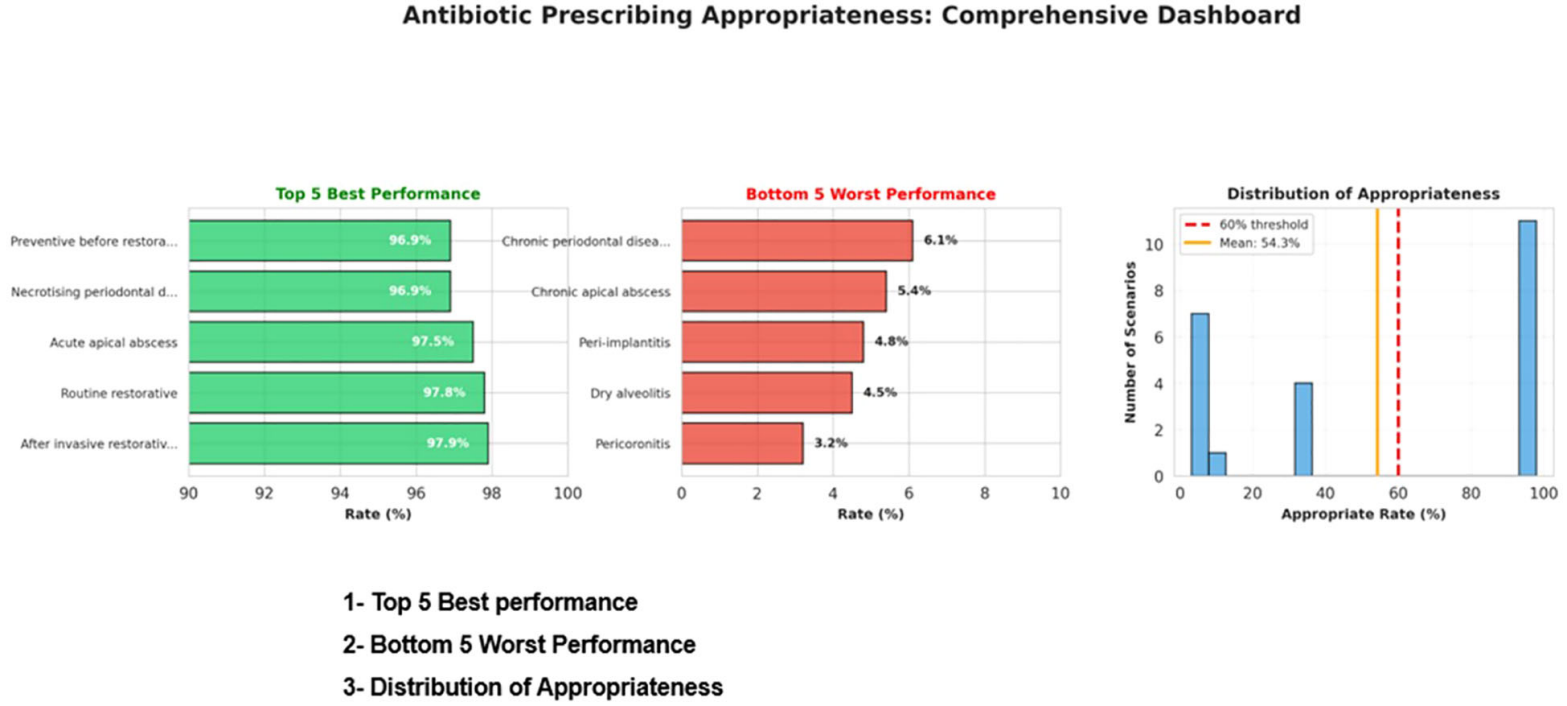
Heatmap of prescribing appropriateness by category. Dental procedures by their indication for antibiotic use and illustrates the proportion of appropriate versus inappropriate prescribing across five main clinical categories: prophylaxis, periodontal, surgical, endodontic, and restorative cases.

**Figure 3:**
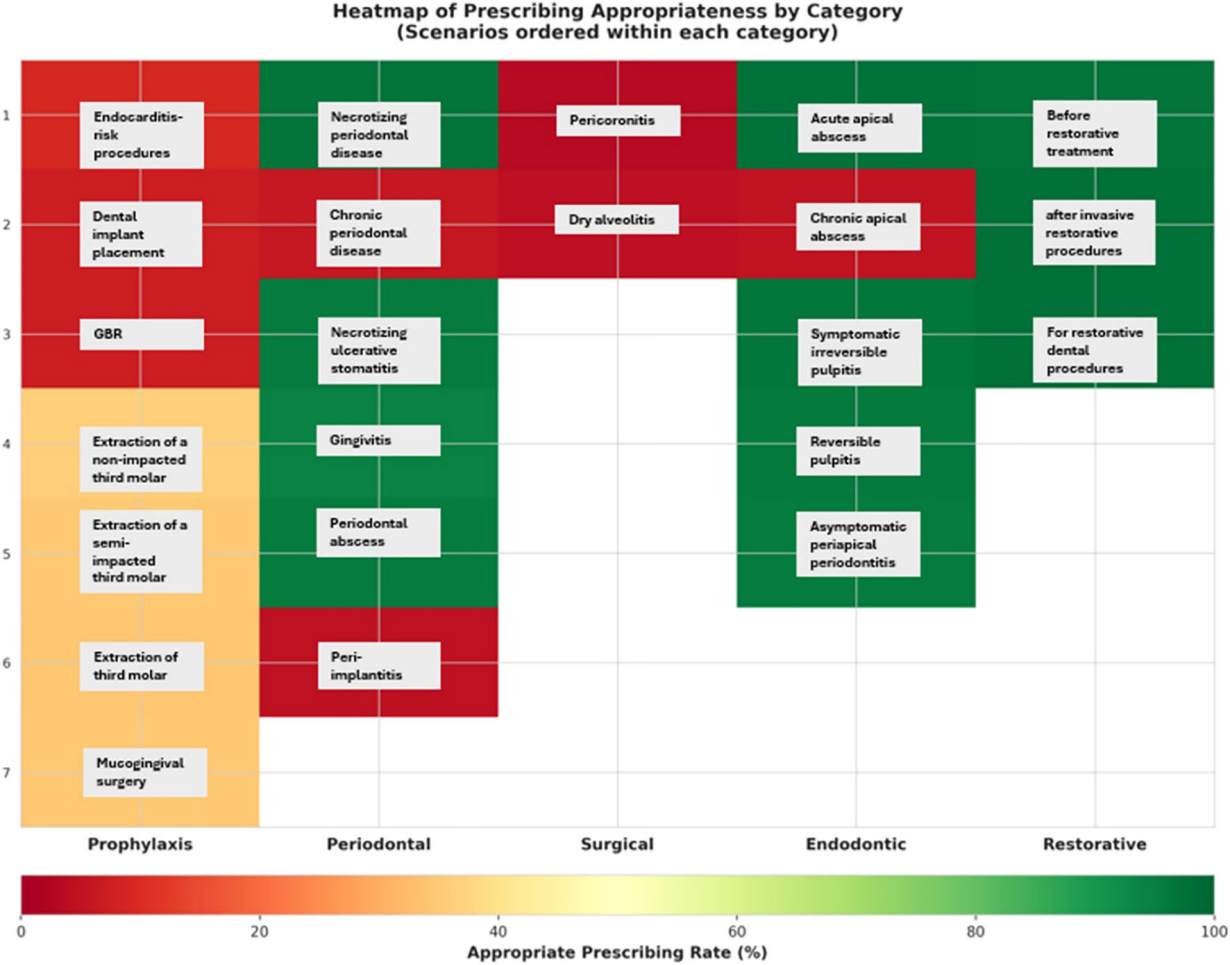
Antibiotic prescribing appropriateness: comprehensive dashboard. Antibiotic prescribing adherence in dental practices and identifies areas for improvement, with Top 5 Best Performance, Bottom 5 Worst Performance and Distribution of Appropriateness.

Results were visualized through diverging bar charts, heatmaps, and dashboard summaries to depict trends in antibiotic prescribing behaviors among Saudi dentists (**Figures 1**–**3**). The visual analyses demonstrated notable variation in prescribing appropriateness across clinical domains, with distinct differences between indicated and non-indicated conditions. The comprehensive dashboard further identified the top and bottom prescribing scenarios and highlighted performance relative to the 60% appropriateness threshold. This integrated presentation provided a clear, evidence-based overview of adherence patterns and key gaps in dental antibiotic stewardship.

### 2.6 Quality assurance and bias control

Several methodological measures were adopted to minimize potential sources of bias and enhance the validity of the study findings. Selection bias was reduced through the widespread dissemination of the survey link across multiple social media platforms such as WhatsApp, X (formerly Twitter), to ensure broad geographic, demographic, and professional representation of dental practitioners across the Kingdom of Saudi Arabia. Participation was entirely voluntary and anonymous, which helped mitigate social desirability bias and encouraged honest and independent reporting of antibiotic prescribing practices and attitudes toward antimicrobial stewardship.

To minimize response bias, the questionnaire was carefully designed with clear, concise, and neutrally worded items to avoid leading or suggestive phrasing. The instrument’s validity and reliability were verified through based on consultants’ recommendations a pilot study involving 10 dental practitioners in Saudi Arabia. Feedback from the pilot phase was incorporated to refine the question wording, improve flow, and optimize survey logic.

To ensure data completeness and accuracy, all items in the online questionnaire were set as mandatory fields, effectively eliminating missing responses. Additionally, we do check within the results to identify and exclude duplicate entries based on IP address and timestamp verification, thereby preventing duplication bias before doing data cleaning.

These methodological safeguards improved the reliability, consistency, and accuracy of the study, ensuring a true reflection of antibiotic prescribing behaviors among dentists in Saudi Arabia.

## 3 Results

### 3.1 Characteristics of participants

A total of 905 practicing dentists participated in and completed the survey. **Table 1** summarizes the characteristics of the study participants. The largest proportion of respondents were aged 31–50 years (366, 40.4%), followed by those aged over 50 years (315, 34.8%) and ≤30 years (224, 24.8%). Nearly two-thirds of the participants were male (595, 65.7%), and over half worked in the public sector (530, 58.6%).

**Table 1:** Characteristics of dentists included in the study (n=905).

Characteristic	Number	Percent
Age (years)		
◼ ≤30	224	24.8%
◼ 31-50	366	40.4%
◼ >50	315	34.8%
Gender		
◼ Male	595	65.7%
◼ Female	310	34.3%
Work sector		
◼ Public	530	58.6%
◼ Private	375	41.4%
First-choice antibiotic class		
◼ Penicillins	342	37.8%
◼ Macrolides	300	33.1%
◼ Cephalosporins	165	18.2%
◼ Fluoroquinolones	98	10.8%
Perceived need for dentistry-specific antibiotic guidelines		
◼ No	168	18.6%
◼ Yes	737	81.4%
Assess plaque control before prescribing antibiotics		
◼ No	855	94.5%
◼ Yes	50	5.5%
Awareness of antibiotic resistance		
◼ No	8	0.9%
◼ Yes	897	99.1%
Climate change is related to antibiotic overuse and resistance		
◼ No	864	95.5%
◼ Yes	41	4.5%
Diet can affect antibiotic resistance		
◼ No	852	94.1%
◼ Yes	53	5.9%

Regarding antibiotic preferences, penicillins were the most frequently selected first-choice antibiotic class (342, 37.8%), followed by macrolides (300, 33.1%) and cephalosporins (165, 18.2%), while fluoroquinolones were the least preferred (98, 10.8%).

A substantial majority of respondents expressed a need for dentistry-specific antibiotic guidelines (737, 81.4%). Only a small proportion reported that they assess plaque control before prescribing antibiotics (50, 5.5%). Awareness of antibiotic resistance was nearly universal (897, 99.1%).

Furthermore, most dentists did not believe that climate change is related to antibiotic overuse and resistance (864, 95.5%). Conversely, 5.9% (53) believed that diet may influence antibiotic resistance.

### 3.2 Antibiotic prescribing practices among dentists

**Table 2** presents participants reported antibiotic prescribing practices and the proportion prescribing antibiotics appropriately according to guideline-supported recommendations (**Supplementary Table 1**).

**Table 2:** Number and percentage of dentists who reported appropriate prescribing of antibiotics (n = 905).

Antibiotic prescription domains	Number	Percent
Antibiotic prophylaxis		
When do you prescribe antibiotic prophylaxis for patients undergoing:		
◼ Endocarditis-risk procedures	81	9.0%
◼ Extraction of a non-impacted third molar	328	36.2%
◼ Extraction of a semi-impacted third molar	316	34.9%
◼ Extraction of third molar, inclusive (all cases)	314	34.7%
◼ Dental implant placement	65	7.2%
◼ Hard tissue augmentation (GBR)	64	7.1%
◼ Mucogingival surgery	317	35.0%
Periodontal cases		
Do you prescribe antibiotic therapy for patients with:		
◼ Necrotizing periodontal disease	877	96.9%
◼ Chronic periodontal disease	55	6.1%
◼ Necrotizing ulcerative stomatitis	865	95.6%
◼ Gingivitis	855	94.5%
◼ Periodontal abscess	866	95.7%
◼ Peri-implantitis	43	4.8%
Surgical cases		
Do you prescribe antibiotic therapy for patients with:		
◼ Pericoronitis	29	3.2%
◼ Dry alveolitis	41	4.5%
Endodontic cases		
Do you prescribe antibiotic therapy for patients with:		
◼ Acute apical abscess	882	97.5%
◼ Chronic apical abscess	49	5.4%
◼ Symptomatic irreversible pulpitis	873	96.5%
◼ Reversible pulpitis	868	95.9%
◼ Asymptomatic periapical periodontitis	864	95.5%
Restorative cases		
Do you prescribe antibiotics:		
◼ As a preventive measure before restorative treatment	877	96.9%
◼ Routinely after invasive restorative procedures	886	97.9%
◼ For routine restorative dental procedures	885	97.8%
^a^ Good antibiotic prescribing practice among participants	358	39.6%

a Good prescribing practice was defined as correctly answering ≥60% of the listed questions (Bloom’s cut-off).

### 3.3 Antibiotic prophylaxis

Overall, dentists demonstrated suboptimal adherence to recommended antibiotic prophylaxis guidelines. Only a small proportion correctly reported prescribing an ultrashort prophylactic regimen (administration 1 hour before surgery) for endocarditis-risk procedures (9.0%), dental implant placement (7.2%), and hard tissue augmentation (GBR) (7.1%).

For procedures where no antibiotic prophylaxis is indicated, approximately one-third of participants correctly selected “no medication,” including extraction of a non-impacted third molar (36.2%), extraction of a semi-impacted third molar (34.9%), all third-molar cases (34.7%), and mucogingival surgery (35.0%).

#### 3.3.1 Periodontal cases

Most dentists demonstrated appropriate prescribing for periodontal conditions where antibiotics are indicated, including necrotizing periodontal disease (96.9%), necrotizing ulcerative stomatitis (95.6%), and periodontal abscess (95.7%). However, for conditions where antibiotics should not be prescribed, correct responses were: 6.1% for chronic periodontal disease, 94.5% for gingivitis, and 4.8% for peri-implantitis.

#### 3.3.2 Surgical cases

Appropriate prescribing for surgical conditions was low. Only 3.2% correctly reported not prescribing antibiotics for pericoronitis, and 4.5% for dry alveolitis, indicating substantial overprescription.

#### 3.3.3 Endodontic cases

Respondents showed good adherence in most endodontic situations requiring antibiotics, such as acute apical abscess (97.5%). High correct-response rates were also observed for conditions where antibiotics are not indicated, including symptomatic irreversible pulpitis (96.5%).

However, only 5.4% correctly recognized that antibiotics should not be prescribed for chronic apical abscess.

#### 3.3.4 Restorative cases

Prescribing practices for restorative procedures were largely appropriate, with nearly all respondents correctly indicating that antibiotics are not required: 96.9% before restorative treatment, 97.9% after invasive restorative procedures, and 97.8% for routine restorative procedures.

### 3.4 Overall quality of prescribing practices

Overall, only 39.6% of participants demonstrated good antibiotic prescribing practice, defined as correctly answering ≥60% of the listed questions (≥14 of 23 items) about antibiotic prescription across various dental clinical scenarios.

Figures Visual representation of antibiotic prescribing practices.

**Figure 1** illustrates a diverging view of appropriateness for indicated versus inappropriateness for non-indicated conditions, highlighting the balance of correct and incorrect antibiotic prescriptions across various dental scenarios.

**Figure 2** presents a heatmap of prescribing appropriateness by category, organizing dental procedures according to their indication for antibiotic use and illustrating the proportion of appropriate versus inappropriate prescribing across five main clinical categories: prophylaxis, periodontal, surgical, endodontic, and restorative cases.

**Figure 3** This figure effectively summarizes the overall adherence to antibiotics prescribing guidelines and identifies areas for improvement in dental practices. It consists of three key components:

1. Top 5 Best Performance: This section highlights the scenarios where antibiotic prescribing was most appropriate like necrotizing periodontal disease, acute apical abscess where it was indicated, as well as preventive measure before restorative treatment, routinely after invasive restorative procedures, and for routine restorative dental procedures, where it was non-indicated, indicating high adherence to guidelines.
2. Bottom 5 Worst Performance: This section showcases the scenarios where antibiotic prescribing was least appropriate, highlighting areas with significant over-prescription like chronic periodontal disease, chronic apical abscess, peri-implantitis, dry alveolitis and pericoronitis.
3. Distribution of Appropriateness: This section visualizes the overall distribution of antibiotic prescribing practices across all scenarios. It displays the number of scenarios at different levels of appropriateness, with the mean appropriateness rate and the 60% threshold line marking the level of adherence considered acceptable according to best practice guidelines. The distribution reveals that many scenarios fall below the recommended threshold, indicating a need for improvement in prescribing practices.

### 3.5 Associations between participant characteristics and quality of antibiotic prescribing practice

Associations between participants’ characteristics and the quality of antibiotic prescribing practice are presented in **Table 3**. The findings show that prescribing quality was significantly associated with age, gender, and perceived need for dentistry-specific antibiotic guidelines (p < 0.05). Dentists in the ≤30 years (102 of 224; 45.5%) and >50 years age groups (132 of 315; 41.9%) demonstrated higher proportions of good prescribing practice compared with those in the 31–50 years group (124 of 366; 33.9%). Female dentists also exhibited significantly better prescribing quality than male dentists, with 152 of 310 (49.0%) demonstrating good practice compared with 206 of 595 (34.6%) among males. In addition, dentists who reported a need for dentistry-specific antibiotic guidelines showed lower levels of good prescribing practice, with 277 of 737 (37.6%) meeting the criteria for good practice compared with 81 of 168 (48.2%) among those who did not perceive such a need.

**Table 3:** Associations between dentists’ characteristics and quality of antibiotic prescribing practices.

Quality of antibiotic prescribing practice/n (%)			
Characteristic	Good practice (n = 358, 39.6%)	Poor practice (n = 547, 60.4%)	*p*
Age (years)			
≤30 (n=224)	102 (45.5%)	122 (54.5%)	0.011*
31-50 (n=366)	124 (33.9%)	242 (66.1%)	
>50 (n=315)	132 (41.9%)	183 (58.1%)	
Gender			
Male (n=595)	206 (34.6%)	389 (65.4%)	<0.001*
Female (n=310)	152 (49.0%)	158 (51.0%)	
Work sector			
Public (n=530)	204 (38.5%)	326 (61.5%)	0.435
Private (n=375)	154 (41.1%)	221 (58.9%)	
Perceived need for dentistry-specific antibiotic guidelines			
No (n=168)	81 (48.2%)	87 (51.8%)	0.011*
Yes (n=737)	277 (37.6%)	460 (62.4%)	

*Denotes significant difference at *p* < 0.05 as indicated by Chi-square statistics.

### 3.6 Predictors of good antibiotic prescribing practice

The results presented in **Table 4** indicate that age, gender, and perceived need for dentistry-specific antibiotic guidelines were significant predictors of good antibiotic prescribing practice (p < 0.05). Compared with dentists aged ≤30 years (reference group), those aged 31–50 years had significantly lower odds of demonstrating good prescribing practice (OR = 0.67; 95% CI: 0.47–0.95; p = 0.025), whereas dentists aged >50 years did not differ significantly (OR = 0.92; 95% CI: 0.65–1.31; p = 0.644).

**Table 4:** Predictors and factors associated with good antibiotic prescribing practice.

Good antibiotic prescribing practice		
Predictors/Associated factors	Odds ratio (95% CI) *p*	
Age (years)		
◼ ≤30	[Reference]	0.025*
◼ 31-50	0.67 (0.47–0.95)	
◼ >50	0.92 (0.65–1.31)	0.644
Gender		
◼ Male	[Reference]	<0.001^*^
◼ Female	1.74 (1.31–2.31)	
Work sector		
◼ Private	[Reference]	0.493
◼ Public	0.91 (0.69–1.20)	
Perceived need for dentistry-specific antibiotic guidelines		
◼ No	[Reference]	0.019*
◼ Yes	0.66 (0.47–0.93)	

The odds ratio and 95% confidence interval were calculated by a multivariate binary logistic model.

*Denotes significant difference at *p* < 0.05.

Female dentists were more likely to exhibit good prescribing practice than male dentists (OR = 1.74; 95% CI: 1.31–2.31; p < 0.001). Additionally, dentists who reported a need for dentistry-specific antibiotic guidelines had significantly lower odds of demonstrating good practice compared with those who did not perceive such a need (OR = 0.66; 95% CI: 0.47–0.93; p = 0.019).

Overall, these findings suggest that younger dentists (≤30 years), female dentists, and those who disagreed with the perceived need for additional antibiotic guidelines tend to exhibit better antibiotic prescribing practices.

## 4 Discussion

This study represents one of the first national-level investigations in Saudi Arabia to assess dental antibiotic prescribing practices through a One Health framework integrating environmental, dietary, and behavioral determinants of antimicrobial resistance (AMR). By examining often-overlooked factors such as climate awareness, plaque assessment, and dietary influences, the research moves beyond traditional clinical considerations. It offers a multidimensional perspective on antibiotic stewardship in dentistry, emphasizing the interconnectedness of human health, environmental conditions, and lifestyle choices in addressing AMR.

### 4.1 Antibiotic prescribing in Saudi dental practice: a misalignment between antimicrobial resistance awareness and clinical practice

This national survey of 905 dentists practicing in Saudi Arabia reveals substantial gaps between awareness of antimicrobial resistance (AMR) and corresponding prescribing behavior. Although 99.1% of participants reported awareness of AMR, only 39.6% demonstrated good prescribing practice (**Tables 1**, **2**). Similar inconsistencies between knowledge and behavior have been previously reported in Saudi dental practice, including studies from Riyadh, Jeddah, and Albaha showing inappropriate antibiotic prescription for non-indicated conditions such as irreversible pulpitis, dry socket, and postoperative pain (Alattas and Alyami, 2017; Alzahrani et al., 2020; Sakka et al., 2023; Méndez-Millán et al., 2024).

A major operational gap identified is that only 5.5% of dentists reported assessing plaque control before prescribing antibiotics (**Table 1**). Given that periodontal and peri-implant diseases are fundamentally biofilm-driven, international S3-level guidelines emphasize mechanical debridement and oral-hygiene reinforcement as first-line therapy (Sanz et al., 2020). Behavioral pressures such as patient expectations may further contribute to unnecessary antibiotic use, as described in qualitative studies (Ramanathan et al., 2024).

A novel feature of this study is its inclusion of climate and diet as emerging determinants of AMR. Despite clear ecological data linking higher ambient temperatures with accelerated spread of resistance (MacFadden et al., 2018), only 4.5% of participants recognized this connection. Similarly, just 5.9% acknowledged that dietary patterns influence AMR, although Western-style, low-fiber, high-fat diets have been shown to increase resistome abundance, and foodborne transmission is well-documented (Keskey et al., 2025). By integrating environmental and behavioral determinants, this study pioneers the application of a One Health approach to dental antibiotic stewardship in Saudi Arabia.

“Penicillins were the most commonly selected first-line antibiotic class, chosen by 37.8% of respondents (**Table 1**), followed by macrolides (33.1%) and cephalosporins (18.2%), reflecting a prescribing trend broadly consistent with international guidelines (Hirayama et al., 2025).

### 4.2 Antibiotic use across dental conditions

The study revealed that only 9.0% of dentists appropriately prescribed ultrashort antibiotic prophylaxis for patients at risk of infective endocarditis—an approach aligned with global recommendations such as those from the American Heart Association (AHA) and the National Institute for Health and Care Excellence (Lockhart et al., 2019). However, many dentists reported prophylactic antibiotic use for procedures where such use is not supported by guidelines. Notably, 36.2%, 34.9%, and 35.0% of respondents accurately recognized that antibiotic prophylaxis was not indicated for non-impacted third molar extractions, semi-impacted third molars, and mucogingival surgery, respectively. In contrast, only 7.2% and 7.1% correctly identified that antibiotics were unnecessary for implant placement and guided bone regeneration respectively, indicating substantial variability in adherence to evidence-based recommendations. This pattern reflects a dual issue: overuse of antibiotics in low-risk dental procedures (Thompson et al., 2021), and underuse in high-risk patients who would benefit from prophylaxis (Thornhill et al., 2022) (**Figure 1**). Such prescribing behavior suggests critical gaps in clinician awareness and implementation of evidence-based guidelines. These findings echo broader international concerns about inappropriate dental antibiotic use (Iyer et al., 2025), and they reinforce the urgent need for national guidelines (Fletcher and Jones, 2025), targeted continuing education (Goff et al., 2020), and audit-feedback interventions to align clinical practice with prophylactic antibiotic standards (Elouafkaoui et al., 2016).

Adherence to guideline-supported antibiotic prescribing was highest in restorative scenarios, such as after invasive or routine restorative procedures, or as a preventive measure before restorative treatment, with the understanding that in these cases, antibiotics may not always be necessary. The second one is endodontic scenarios, such as reversible and irreversible pulpitis without systemic symptoms. These conditions typically do not require antibiotics, as inflammation is not due to systemic bacterial infection and is best managed with operative treatment. In contrast, adherence was markedly lower in prophylaxis and minor surgical cases: endocarditis-risk procedures (9.0%), dental implant placement (7.2%) and GBR (7.1%) as prophylaxis, and pericoronitis (3.2%), and dry socket (4.5%) as surgical cases (**Table 2**, **Figure 2**). Clinical guidelines explicitly advise against the prescribing of antibiotics in these conditions unless systemic involvement is present. For example, dry socket (alveolar osteitis) is a localized post-extraction inflammation best treated with local measures such as irrigation and analgesics. Similarly, pericoronitis is often self-limiting and responds to local debridement and improved oral hygiene unless there are signs of spreading infection. As illustrated in **Figure 3**, antibiotic prescribing appropriateness varied widely across clinical scenarios, with adherence exceeding 95% in acute and restorative cases, but falling below 10% in chronic and post-surgical conditions.

These findings are consistent with international clinical guidelines and systematic reviews that strongly discourage the use of antibiotics for managing pulpitis, as the condition is primarily inflammatory rather than infectious. Similarly, the results for dry socket and localized pericoronitis align with current evidence indicating that antibiotic therapy is not warranted in the absence of systemic involvement (Baskaradoss et al., 2018; Lockhart et al., 2019; Tampi et al., 2019; Schmidt et al., 2021).

These findings on pericoronitis and dry socket do not align with the studies by Lockhart PB et al., Baskaradoss JK et al., Schmidt J et al., and Tampi MP et al., which recommend against antibiotic use for pericoronitis unless there are systemic signs of infection. Our results suggest a different approach, where antibiotics may be considered even in the absence of systemic involvement. In contrast, the findings of pulpitis align with international clinical guidelines and reviews that consistently discourage antibiotic use for pulpitis (Baskaradoss et al., 2018; Lockhart et al., 2019; Tampi et al., 2019; Schmidt et al., 2021).

### 4.3 Associated factors and predictors of good antibiotic prescribing

The analysis of **Tables 3** and **4** reveals key behavioral and demographic associated factors and predictors influencing antibiotic prescribing practices among dentists in Saudi Arabia.

Dentists aged ≤30 years demonstrated higher adherence to prescribing guidelines compared to older counterparts, suggesting a positive effect of recent training in antimicrobial stewardship. This aligns with international findings where younger clinicians often perform better due to exposure to modern curricula (Salako et al., 2004).

Female dentists also exhibited significantly better prescribing behavior than males a pattern consistent with broader evidence showing that women tend to be more guideline-compliant and cautious in clinical decision-making (Halboub et al., 2016).

Interestingly, no significant difference was found between public and private sector dentists, reinforcing that inappropriate prescribing is a system-wide issue rather than one confined to institutional type (Rodríguez-Fernández et al., 2023).

A particularly revealing finding was that dentists who agreed with the perceived need for additional antibiotic guidelines were less likely to prescribe appropriately. This may reflect a self-acknowledged gap in knowledge or a lack of access to structured guidance. The role of clear, professional-specific guidelines has been widely recognized as essential to improving antibiotic use in dentistry.

### 4.4 Limitations

This study presents valuable insights into antibiotic prescribing practices among dentists in Saudi Arabia, yet it has several limitations that warrant consideration. First, the findings are based entirely on self-reported data, which are inherently prone to social desirability bias respondents may have overstated their adherence to guidelines to appear more compliant. Furthermore, the cross-sectional nature of the study captures practices at a single point in time and therefore limits the ability to draw causal inferences or observe trends over time. The absence of clinical validation such as matching survey responses with actual prescription records also limits the accuracy of reported behaviors.

Additionally, voluntary participation may have introduced selection bias, potentially overrepresenting dentists with stronger interest or awareness in antimicrobial stewardship. The scope of the survey, while inclusive of common procedures, did not encompass all clinical scenarios where antibiotics are prescribed, nor did it assess critical parameters such as dosage, duration, or awareness of local resistance patterns. While the survey addressed perceived needs and attitudes, it did not evaluate dentists’ actual clinical decision-making skills through case-based scenarios or knowledge tests. Finally, although the sample was drawn from across the country, regional differences in practice settings, access to continuing education, and policy implementation may affect the generalizability of the results beyond the study population. Future research should use longitudinal or mixed-method designs linking survey data with actual prescription records to assess real-world behaviors. Interventional and qualitative studies are also needed to evaluate stewardship programs, digital prescribing tools, and One Health–based educational initiatives to promote sustainable antibiotic use in dentistry. Additionally, this study did not stratify participants by dental specialty, which limited the ability to conduct specialty-specific subgroup analyses or include specialty in regression models. Considering the variation observed across clinical domains, future studies should incorporate dental specialty as a key variable to enable more detailed analysis and support targeted stewardship strategies.

The extended data collection period (April 2024–February 2025) may have introduced temporal bias, as prescribing behaviors could have evolved during the study timeframe. Additionally, reliance on self-reported practices introduces potential social desirability and recall bias. The lack of validation against actual prescription records further limits confirmation of real-world prescribing behavior.

## 5 Conclusion

This study highlights a critical disconnect between antimicrobial awareness and actual prescribing behavior among dentists in Saudi Arabia. While most participants acknowledged the global threat of antimicrobial resistance, a substantial proportion continued to prescribe antibiotics inappropriately, particularly for conditions where local therapy would suffice. Predictors such as younger age and female gender were associated with better adherence to guidelines, whereas expressed need for prescribing guidance correlated with poorer practice, suggesting uncertainty and lack of structured support. The findings indicate limited awareness of broader One Health–related determinants of antimicrobial resistance, including climate and dietary factors. As this study assessed awareness rather than direct impact on prescribing behavior or resistance outcomes, these observations should be interpreted as exploratory. Further research is needed to evaluate whether and how environmental and behavioral determinants may influence dental prescribing practices and antimicrobial resistance patterns. To strengthen dental antibiotic stewardship, there is an urgent need for national guidelines, continuous professional education, and feedback-driven interventions that align daily practice with evidence-based standards.

## Data Availability

The raw data supporting the conclusions of this article will be made available by the authors, without undue reservation.
