# Supplementary material for: From prescription to resistance: integrating one health perspectives into dental antibiotic stewardship in Saudi Arabia
Source: Front Cell Infect Microbiol. 2026 Mar 25;16:1781988. doi: 10.3389/fcimb.2026.1781988 (PMC13057380; doi:10.3389/fcimb.2026.1781988)
Supplement: Supplementary file 1 [file Table1.docx]

**Supplementary Table 1: Guideline-supported responses for antibiotic prescription**

| Antibiotic prescription domains | Guideline-supported response |
| --- | --- |
| Antibiotic prophylaxis |  |
| When do you prescribe antibiotic prophylaxis for patients undergoing: |  |
| - Endocarditis-risk procedures | Ultrashort: administration 1 h before surgery. |
| - Extraction of a non-impacted third molar | I do not prescribe any medication in this case. |
| - Extraction of a semi-impacted third molar | I do not prescribe any medication in this case. |
| - Extraction of third molar, inclusive (all cases) | I do not prescribe any medication in this case. |
| - Dental implant placement | Ultrashort: administration 1 h before surgery. |
| - Hard tissue augmentation (GBR) | Ultrashort: administration 1 h before surgery. |
| - Mucogingival surgery | I do not prescribe any medication in this case. |
| Periodontal cases |  |
| Do you prescribe antibiotic therapy for patients with: |  |
| - Necrotising periodontal disease | Yes |
| - Chronic periodontal disease | No |
| - Necrotising ulcerative stomatitis | Yes |
| - Gingivitis | No |
| - Periodontal abscess | Yes |
| - Peri-implantitis | No |
| Surgical cases |  |
| Do you prescribe antibiotic therapy for patients with: |  |
| - Pericoronitis | No |
| - Dry alveolitis | No |
| Endodontic cases |  |
| Do you prescribe antibiotic therapy for patients with: |  |
| - Acute apical abscess | Yes |
| - Chronic apical abscess | No |
| - Symptomatic irreversible pulpitis | No |
| - Reversible pulpitis | No |
| - Asymptomatic periapical periodontitis | No |
| Restorative cases |  |
| Do you prescribe antibiotics: |  |
| - As a preventive measure before restorative treatment | No |
| - Routinely after invasive restorative procedures | No |
| - For routine restorative dental procedures | No |
